# Recent advances in understanding biliary atresia

**DOI:** 10.12688/f1000research.16732.1

**Published:** 2019-02-25

**Authors:** Andrew Wehrman, Orith Waisbourd-Zinman, Rebecca G Wells

**Affiliations:** 1 Gastroenterology, Hepatology, and Nutrition, The Children's Hospital of Philadelphia, Philadelphia, PA, 19104, USA; 2 Gastroenterology, Hepatology, and Nutrition, Schneider Children's Medical Center of Israel, Petach-Tiqva, Israel; 3 Medicine and Bioengineering, University of Pennsylvania, Philadelphia, PA, 19104, USA

**Keywords:** bile duct, Kasai, hepatoportoenterostomy, bilirubin

## Abstract

Biliary atresia (BA) is a neonatal liver disease characterized by progressive obstruction and fibrosis of the extrahepatic biliary tree as well as fibrosis and inflammation of the liver parenchyma. Recent studies found that infants who will go on to develop BA have elevated direct bilirubin levels in the first few days of life, suggesting that the disease starts *in utero*. The etiology and pathogenesis of BA, however, remain unknown. Here, we discuss recent studies examining potential pathogenetic mechanisms of BA, including genetic susceptibility, involvement of the immune system, and environmental insults such as viruses and toxins, although it is possible that there is not a single etiological agent but rather a large group of injurious insults that result in a final common pathway of extrahepatic bile duct obstruction and liver fibrosis. The management and diagnosis of BA have not advanced significantly in the past decade, but given recent advances in understanding the timing and potential pathogenesis of BA, we are hopeful that the next decade will bring early diagnostics and novel therapeutics.

## Introduction

**Figure 1.**
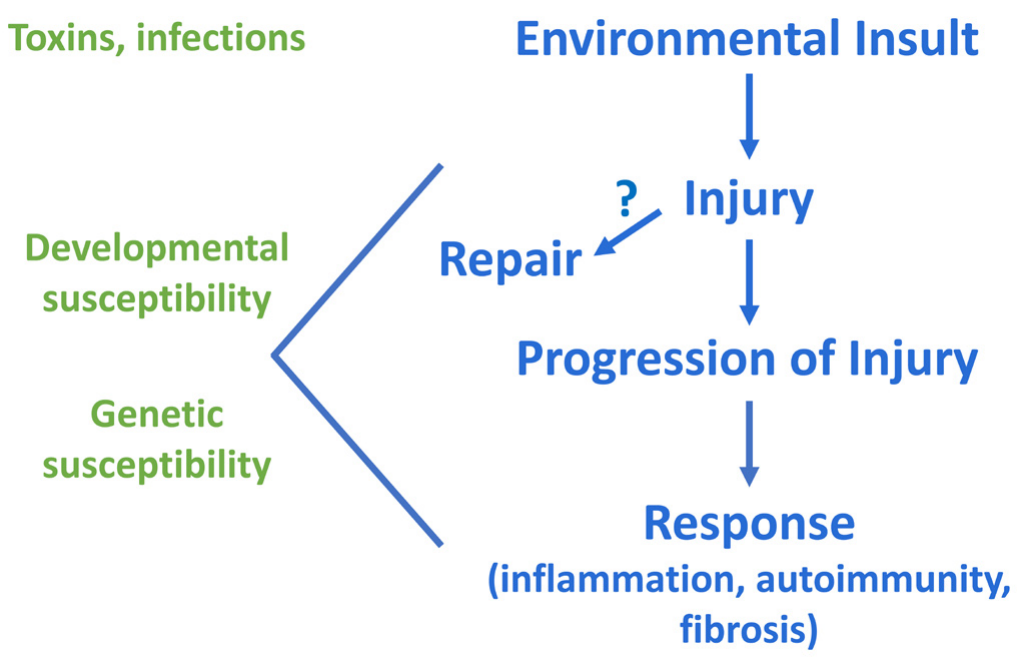
Schematic of hypothesis of biliary atresia etiology. Right side shows potential sequence of events, beginning with a prenatal bile duct injury, in biliary atresia. Left side highlights the potential contribution of developmental and genetic susceptibility to the injury and response.

Biliary atresia (BA) is a fibrotic disease affecting primarily the extrahepatic biliary tree that presents exclusively in infants. Children appear normal at birth but rapidly develop progressive liver fibrosis, bile duct obstruction, and cholestasis over the first several months of life. Surgical management with a Kasai hepatoportoenterostomy (HPE) can relieve the obstruction and, in about 50% of infants in North America, lead to bile drainage; however, in most children, there is ongoing fibrosis and inflammation in the liver, leading to end-stage liver disease and the need for liver transplant during childhood ^1^. There may be geographic differences in the success of HPE, with successful drainage shown as high as 70% in a Japanese registry, depending on the age at time of surgery ^2^. BA remains the leading indication for pediatric liver transplantation, and, to date, no medical interventions have been identified. The etiology of the disease is unknown, adding to the difficulties in studying and developing therapies for BA. Recent data, however, have changed our understanding of the disease. A hypothesis supported by current data proposes that a prenatal environmental insult (toxin or virus) damages the extrahepatic bile duct in a genetically and developmentally susceptible fetus but spares the mother and that injury progresses after birth, leading to immune system activation and possible autoimmunity ( Figure 1). This review will discuss literature supporting this hypothesis on the etiology of BA as well as recent studies involving clinical management and prognosis after HPE in BA.

## Biliary atresia likely begins *in utero*

Fundamental to this hypothesis are recent studies showing that conjugated (or direct) bilirubin is abnormal at birth in children who go on to develop BA. Harpavat *et al*. studied over 10,000 infants who were screened with fractionated bilirubin in the first 72 hours of life ^3, 4^. Those with abnormal direct or conjugated bilirubin at birth underwent measurement of fractionated bilirubin again 2 weeks later. Of the 13 infants who tested positive on the second screen, two had BA, and no infants with BA were missed ^3, 4^. These data and similar studies from the same group provide convincing evidence that the initial injury in BA occurs before birth and suggests that the fetal bile duct is uniquely susceptible to injury and fibrotic sequellae. Understanding the timing of the initial injury in BA will be important in directing future research into factors that lead to injury or repair of the neonatal bile duct; regardless, this study raises the possibility that diagnosis and treatment of newborns (shortly after birth) may be both possible and preferable compared with treatment at the usual time of HPE.

## Potential etiologies of biliary atresia

Epidemiology studies suggest that the cause of BA is environmental ^5^, and both infections and environmental toxins have been studied as potential agents causing damage leading to BA. One heavily studied infectious agent is rotavirus. Early postnatal infection of mouse pups with rhesus rotavirus (RRV) leads to a BA-like inflammatory response in the extrahepatic bile duct and has been widely used as a model of BA; however, rotavirus has not been shown to cause human BA, and a recent study showed that the incidence of BA has not changed despite widespread rotavirus vaccination ^6^. Cytomegalovirus (CMV) DNA was identified in 60% of liver biopsies from children with BA in China ^7^, and in a large study of 210 infants with BA in the UK, 9.5% of infants were CMV IgM–positive at presentation; those who were positive had worse outcomes, including decreased survival with a native liver ^8^. CMV may be a trigger for many infants who develop BA; however, validation of the data in additional centers is required.

An isoflavonoid biliary toxin called biliatresone was recently isolated from Australian plants after several large-scale outbreaks of a BA-like disease in newborn livestock born to mothers that grazed on these plants while pregnant ^9^. Biliatresone acts a biliary toxin in *in vitro* organoid models, mouse extrahepatic bile duct explants, and larval zebrafish, providing a proof of concept that toxins can cause selective extrahepatic biliary damage. Biliatresone treatment leads to luminal obstruction and loss of cholangiocyte polarity in mammalian models ^10^. It appears to injure cholangiocytes in part through depletion of glutathione, and restoration of glutathione stores with *N*-acetyl-L-cysteine mitigates its toxicity in mouse cholangiocytes and zebrafish ^10, 11^. Although biliatresone is not ingested by humans and is unlikely to be the cause of human BA, it is useful as a model for studying toxic insults to neonatal bile ducts and pathways of injury and repair in the neonatal bile duct. It is possible that there are similar environmental toxins that lead to human disease; additionally, understanding the mechanisms whereby biliatresone accumulates in the bile ducts may be important in understanding other potential biliary toxins.

The fact that BA does not occur in humans as discrete outbreaks suggests that there is not a single etiological agent but that there may be a large group of etiological agents, potentially including toxins and infectious agents. Identifying common mechanisms of injury and repair may be a better approach to developing therapeutic agents than searching for single etiological agents.

## Genetic susceptibility in biliary atresia

BA is not a primary genetic disease, although multiple genes that might increase susceptibility to BA have been identified. A genome-wide association study (GWAS) in Chinese infants with BA identified variants in the ADD3 gene; knockdown of ADD3 in zebrafish resulted in intrahepatic biliary abnormalities due to increased hedgehog signaling ^12, 13^. Variants in GPC1 have also been identified in children with BA. Knockdown of GPC1 in zebrafish led to biliary abnormalities, and partial recovery was achieved using a hedgehog antagonist ^14^. A recent GWAS in children with both isolated BA and BA splenic malformation syndrome (BASM) identified the candidate gene EFEMP1, which encodes the extracellular matrix (ECM) protein fibulin-3 and may be important in both the structure and repair of the ECM ^15^. Preliminary work via whole exome sequencing of family trios has identified a variant in the primary cilia protein PKD1L1, suggesting that primary cilia may also play a role in the susceptibility of the extrahepatic bile duct to injury ^16^. Overall, there appear to be multiple gene defects associated with BA, but all appear to increase susceptibility or modify the phenotype rather than being primarily responsible for injury.

The lack of an identifiable genetic cause of BA has led to the hypothesis that maternal microchimerism (postzygotic somatic mutation) may be part of the etiology ^17, 18^. Although a recent study has shown no evidence of maternal microchimerism in lymph nodes ^19^, additional studies would be required to both demonstrate the presence of microchimerism in BA and show that it has a causal role in the disease.

## The role of inflammation and autoimmunity

Regardless of the initial injury in BA, a hallmark of the disease is significant inflammation and fibrosis of both the liver and ducts. Abnormalities in innate immunity, cellular immunity, and antibody-mediated immunity have been identified in both human samples and mouse models ^5, 16, 20, 21^. Interleukin 17a (IL17a) has recently been shown to promote macrophage recruitment through chemokine signaling in the RRV mouse model and may be important in the progression of the liver and duct injury ^22^. RRV-infected mice have increased hepatic IL17a mRNA and IL17a antibody treatment mitigated injury ^23^, and children with BA had a higher load of IL17a-positive cells in liver samples compared with both normal and cholestatic controls ^22^. There is indirect evidence that autoimmunity plays a role in the pathogenesis of BA ^16^. In the RRV model of BA, adoptive transfer of T cells from a mouse with BA to a T cell–deficient mouse produces bile duct injury ^24^. Also, in the RRV mouse model, B cell–deficient mice do not develop BA and adoptive transfer of B cells into RRV-infected B cell–deficient mice leads to biliary epithelial damage and T-cell activation, likely mediated through cytokines ^25^. Notably, anti-inflammatory treatments, including steroids ^26^ and intravenous immunoglobulin (IVIG) ^27^, given at the time of HPE, have all failed to change the progression of fibrosis and inflammation.

## Screening and diagnosis

The timing and accuracy of BA diagnosis are highly clinically relevant issues given that older age (>30 days) and greater degree of fibrosis at the time of HPE are associated with increased need for liver transplant ^28– 30^. Stool cards are widely used in certain countries and have been shown to decrease the number of late referrals for evaluation of symptomatic infants and to decrease the age at HPE ^31^. Screening newborns for elevated direct or conjugated bilirubin, as discussed above, offers the potential to identify asymptomatic babies very early in the course of the disease but has relatively low specificity and has not yet been implemented widely ^3, 4^.

The diagnosis of BA in a jaundiced infant can be difficult. An intraoperative cholangiogram remains the gold standard, and frequently a liver biopsy is obtained to aid in diagnosis. A large study of 227 liver biopsies in infants with neonatal cholestasis defined significant features suggestive of BA, including bile plugs, moderate to marked ductular reaction, and portal stromal edema; however, the overall sensitivity and specificity of needle biopsy in the diagnosis of BA were 88.4% and 92.7%, respectively. No findings on liver biopsy were predictive of successful drainage ^28^. A hepatobiliary iminodiacetic acid (HIDA) scan is used in some centers to assess bile passage to the gut, whereas in other centers this is not considered necessary in the setting of a suggestive liver biopsy. Endoscopic retrograde cholangiopancreatography ^32^ and transient elastography ^33^ are being investigated for the diagnosis of BA, but the use of these imaging methods is not widespread. A large proteomics study identified increased matrix metalloproteinase 7 (MMP-7) (expressed by cholangiocytes and released upon injury) in infants with BA as a potential biomarker for BA ^34^. Children with BA have been shown to have elevated serum MMP-7 compared with both healthy and cholestatic controls, and the level of MMP-7 correlated with fibrosis stage ^35^. MMP-7 has been shown to have a high sensitivity and specificity (98.67% and 95%, respectively) when used to distinguish BA from other causes of neonatal cholestasis ^36^. This work has promising implications as a non-invasive biomarker to aid in the diagnosis of BA in the future but is not yet used clinically.

## Clinical management

The management of BA has not changed significantly in the past decade. Most children with diagnosed BA undergo HPE, whereby the atretic extrahepatic bile duct is resected to the level of the porta hepatis and drained via a roux-en-Y loop of jejunum ^37^. HPE is successful in about 50% of infants, and success (indicating bile drainage) is defined as total bilirubin of less than 2 mg/dL at 3 months post-HPE. Children with total bilirubin of more than 2 mg/dL are more likely to have complications of liver disease, including ascites, thrombocytopenia, and decreased survival with native liver, compared with children with adequate drainage ^1^. Bile drainage after HPE, though necessary for transplant-free survival, is not curative, and up to 66% of children with successful HPE will still have ongoing liver fibrosis and portal hypertension ^38^. Thus far, no post-HPE adjuvant therapy has been shown to change outcomes in BA. Several trials showed that prednisone treatment after HPE did not promote improved bile drainage and caused significant adverse effects ^26, 39, 40^. Another trial showed that IVIG administration was similarly ineffective at promoting bile drainage post-HPE ^27^. Supportive therapy to ensure adequate nutrition and supplementation with fat-soluble vitamins remain the mainstay of therapy. Ursodeoxycholic acid is given in the vast majority of the centers, and most centers also prescribe prophylactic antibiotics because of the risk of cholangitis in the first 6 to 12 months of life. All children with BA are at risk for neurodevelopmental delays, but those with unsuccessful HPE have four times the risk of both mental/cognitive/language delays and physical/motor delays ^41^. BA remains the leading indication for liver transplant in pediatric recipients, accounting for 32.3% of pediatric liver transplants in 2016 ^42^.

## Conclusions and future directions

Recently, there has been significant progress in understanding the time course of BA and range of potential etiologies. Solid evidence suggests that a prenatal insult, either toxic or infectious, causes injury in a genetically susceptible fetus, leading to progressive fibro-inflammatory damage to the extrahepatic bile duct and associated immune and autoimmune dysfunction. Current therapies for BA all begin at the time of HPE, which may be too late to significantly change the progression of the disease. New studies showing that asymptomatic infants who will go on to develop BA can be identified weeks to months earlier than the usual time of HPE raise the possibility that the disease can be mitigated before significant damage occurs and potentially cured. Future research and clinical efforts should focus on early diagnosis and on studying the effects of therapeutic agents started within the first few weeks of life.
